# Lipid droplet accumulation in Wdr45-deficient cells caused by impairment of chaperone-mediated autophagic degradation of Fasn

**DOI:** 10.1186/s12944-024-02088-y

**Published:** 2024-03-28

**Authors:** Qiuhong Xiong, Huimin Sun, Yanlin Wang, Qian Xu, Yu Zhang, Mei Xu, Zhonghua Zhao, Ping Li, Changxin Wu

**Affiliations:** Institutes of Biomedical Sciences, Shanxi Provincial Key Laboratory for Medical Molecular Cell Biology, Key Laboratory of Chemical Biology and Molecular Engineering of Ministry of Education, Shanxi University, Taiyuan, 030006 China

**Keywords:** BPAN, Wdr45, Lipid droplet, Accumulation, CMA, Fasn

## Abstract

**Background:**

β-Propeller protein-associated neurodegeneration (BPAN) is a genetic neurodegenerative disease caused by mutations in *WDR45*. The impairment of autophagy caused by WDR45 deficiency contributes to the pathogenesis of BPAN; however, the pathomechanism of this disease is largely unknown. Lipid dyshomeostasis is involved in neurogenerative diseases, but whether lipid metabolism is affected by Wdr45 deficiency and whether lipid dyshomeostasis contributes to the progression of BPAN are unclear.

**Methods:**

We generated Wdr45 knockout SN4741 cell lines using CRISPR‒Cas9-mediated genome editing, then lipid droplets (LDs) were stained using BODIPY 493/503. Chaperone-mediated autophagy was determined by RT-qPCR and western blotting. The expression of fatty acid synthase (Fasn) was detected by western blot in the presence or absence of the lysosomal inhibitor NH_4_Cl and the CMA activator AR7. The interaction between Fasn and HSC70 was analyzed using coimmunoprecipitation (Co-IP) assay. Cell viability was measured by a CCK-8 kit after treatment with the Fasn inhibitor C75 or the CMA activator AR7.

**Results:**

Deletion of Wdr45 impaired chaperone-mediated autophagy (CMA), thus leading to lipid droplet (LD) accumulation. Moreover, Fasn can be degraded via CMA, and that defective CMA leads to elevated Fasn, which promotes LD formation. LD accumulation is toxic to cells; however, cell viability was not rescued by Fasn inhibition or CMA activation. Inhibition of Fasn with a low concentration of C75 did not affect cell viability but decreases LD density.

**Conclusions:**

These results suggested that Fasn is essential for cell survival but that excessive Fasn leads to LD accumulation in Wdr45 knockout cells.

**Supplementary Information:**

The online version contains supplementary material available at 10.1186/s12944-024-02088-y.

## Introduction

β-Propeller protein-associated neurodegeneration (BPAN, OMIM 300,894), previously known as static encephalopathy of childhood with neurodegeneration in adulthood (SENDA), was first described by Gregory in 2009 [1]. BPAN patients are characterized by global developmental delay in early childhood with essentially static, slow motor and cognitive gains until adolescence or early adulthood. In young adulthood, affected individuals develop progressive dystonia, parkinsonism, extrapyramidal signs, and dementia resulting in severe disability [2]. BPAN is caused by mutations in the autophagy-related gene *WDR45* (also known as *WIPI4*) [3], which functions in autophagosome formation [4, 5]. Therefore, impaired autophagy contributes to the pathogenesis of BPAN [6, 7]. However, the pathophysiology of this disease has not been fully elucidated.

Lipids account for more than half of the human mass, but little is known about lipid metabolism in the brain in individuals with health and disease [8, 9]. To date, abnormal accumulation of lipid droplets (LDs) has been observed in neurodegenerative disorders, but whether LD accumulation is neuroprotective or toxic is still unclear [9, 10]. Several studies have demonstrated the protective action of some lipid species, such as n-3 polyunsaturated fatty acid (PUFA) and neuroactive steroids [11–14]. LDs are thought to protect cells from fatty acid toxicity [15]. However, accumulating evidence suggests that LD-related lipotoxicity might occur in neurodegenerative diseases. It has been shown that inhibiting the synthesis of cholesterol by lovastatin and pravastatin in humans markedly reduces the prevalence of Alzheimer’s disease (AD) [16]. In a mouse AD model, increased LDs in ependymal cells suppressed neural stem cell proliferation, and inhibition of oleic acid signaling or synthesis rescued defects in neural stem cells [17]. Several studies have revealed that LD accumulation may contribute to Parkinson’s disease (PD) [18–21]. α-Synuclein can be located on the surface of LDs, and mutated α-synuclein has increased binding affinity to LDs; therefore, LD accumulation promotes the aggregation of α-synuclein [22]. Inhibiting stearoyl-CoA desaturase (SCD) to reduce the levels of unsaturated membrane lipids ameliorated the cytotoxicity of α-synuclein [23]. Taken together, these studies suggest that abnormal lipid metabolism is associated with brain pathology and neurodegeneration. However, whether LDs accumulate and whether abnormal lipid metabolism are involved in BPAN remain to be elucidated.

LDs can be degraded via macroautophagy which is called lipophagy [24]. To date, three types of autophagic pathways, macroautophagy, microautophagy and chaperone-mediated autophagy (CMA), are thought to drive LD degradation [25]. Microlipophagy was first detected in yeast, but in mammals, it was detected only in murine hepatocytes; little is known about the mechanism of this pathway [26]. As WDR45 functions in autophagosome formation, WDR45 deficiency should impair macrolipophagy. CMA can be activated when macroautophagy is impaired [27, 28], and CMA is involved in LD breakdown through the degradation of PLIN2 and PLIN3, which surround and stabilize LDs [29]. However, whether CMA dysregulates lipid metabolism in Wdr45 deletion neurons is still unknown.

In addition to defects in lipolysis and lipophagy, increased lipid biosynthesis also contributes to the accumulation of LDs during neurodegeneration. In yeast, Drosophila and human neuroblastoma cells, the overexpression of α-synuclein causes LD accumulation which may in turn promote the aggregation of α-synuclein [19, 22, 30]. Fatty acid synthase (FASN) catalyzes the synthesis of long-chain fatty acids from acetyl-CoA and malonyl-CoA and is highly expressed in most human cancers, such as breast, lung and prostate cancers [31]. In an AD mouse model, Fasn was elevated, suggesting that Fasn may contribute to LD accumulation in AD mice [32]. However, whether FASN contributes to the pathogenesis of BPAN remains to be addressed.

This study evaluated the activity of CMA, LD accumulation and the function of Fasn in LD accumulation using a Wdr45 knockout (KO) cell line SN4741, which was derived from mouse dopaminergic neurons. The goals of this study were to define the contribution of Wdr45 to lipid homeostasis, and to investigate the mechanism of LD accumulation.

## Materials and methods

### Chemical reagents and antibodies

The following reagents and antibodies were used in this study: oleic acid (OA) (Sigma, O1008); AR7 (TargetMol, T3955); C75 (TargetMol, T10657); BODIPY 493/503 (TargetMol, T36957); Wortmannin (TargetMol, T6283); LysoTracker Green (Beyotime, C1047S); anti-Wdr45 (Proteintech, 19,194, 1:1000); anti-Hsc70 (ABclonal, A14001, 1:1000); anti-Fasn (Proteintech, 10624-2-AP, 1:5000); anti-Lamp2a (Abcam, ab18528, 1:1000); anti-Plin2 (Proteintech, 15,294, 1:5000); anti-GFP (ABclonal, AE012, 1:3000); anti-β-Actin (Absin, abs125702, 1:30000); anti-p62 (Abcam, ab56416, 1:2000); anti-Lc3 (Abcam, ab51520, 1:3000); anti-mouse IgG conjugated with peroxidase (POD) (Proteintech, SA00001-1, 1:10000); anti-rabbit IgG conjugated with POD (Proteintech; SA00001-2, 1:10000); IRDye® 800CW goat anti-mouse IgG (LI-COR Biosciences, 926–32,210, 1:25000) and IRDye® 680RD goat anti-rabbit IgG (LI-COR Biosciences, 926–68,071, 1:25000).

### Generation of Wdr45 knockout SN4741 cell lines

Wdr45 was deleted in SN4741 cells by transient transfection of cells with the pSpCas9(BB)-2 A-Puro (PX459) V2.0 plasmid as described previously [33]. Briefly, the top and bottom strands of the sgRNA were synthesized, and then denatured at 95℃ for 5 min in a water bath and slow cooling at room temperature to annealing. The PX459 vector (a gift from Feng Zhang, Addgene plasmid # 62,988) was linearized by the restriction enzyme BbsI (Thermo Fisher Scientific, ER1012), after which the annealed oligos were ligated into the plasmid by T4 DNA ligase (Thermo Fisher Scientific, EL0012). The constructs were transfected into SN4741 cells using Lipofectamine 3000 (Thermo Fisher Scientific, L3000075) according to the manufacturer’s instructions. After 36 h of transfection, the cells were selected using puromycin at a dose of 1 μg/ml (Solarbio, P8230) for 2 days. The single-cell clones were sorted using a MoFlo Astrios Cell Sorter (Beckman Coulter). Colony-PCR was performed and the PCR products were subsequently sequenced (Sangon Biotech). The gRNAs and PCR primers were listed in Table 1.

**Table 1:** Oligos used in this study

Oligo name	Sequence	Reference
gRNA1 top	CACCGATGACTCAGCAGCCACTTCG	This study
gRNA1 bottom	AAACCGAAGTGGCTGCTGAGTCATC	
gRNA2 top	CACCGTGACACTCGGGACAACCCCA	[34]
gRNA2 bottom	AAACTGGGGTTGTCCCGAGTGTCAC	
gRNA3 top	CACCGAAGCAGCTGCTCGTGTTTCC	[35]
gRNA3 bottom	AAACGGAAACACGAGCAGCTGCTTC	
Wdr45 sequencing 1 F	GATACTCTGAGGTATCCTCCAC	This study
Wdr45 sequencing 1 R	GGAATAGGGTGTCAGGAGAGG	
Wdr45 sequencing 2 F	CGGAAGCAAGTGGTTGAGATCC	This study
Wdr45 sequencing 2 R	CAGGGAAGAGGAACTGACTGTG	
Wdr45 sequencing 3 F	GAGCAGAGCTTACTGCAAAGCC	This study
Wdr45 sequencing 3 R	GCTGCAAGACAGACCCGTAATG	

### Cell culture, transient transfection and compound treatment

SN4741 and HEK293T cells were cultured in Dulbecco’s Modified Eagle Medium (DMEM, BOSTER, PYG0073) supplemented with 10% fetal bovine serum (FBS, BioChannel, China BC-SE-FBS08) and 1% penicillin/streptomycin (Leagene, CA0075) at 37℃ and 5% CO_2_. HEK293T cells were transiently transfected with GFP-HSC70 [36] using Lipofectamine 3000 (Thermo Fisher Scientific, L3000075) according to the manufacturer’s instructions. SN4741 wild-type (WT) and Wdr45 knockout (KO) cells were grown in the presence or absence of OA (300 μM, 24 h); the autophagy inhibitor, Wortmannin (500 nM, 24 h); the lysosome inhibitor, NH_4_Cl (5 mM, 24 h); the CMA activator, AR7 (20 μM, 24 h) or the FASN inhibitor, C75 (10 or 20 μM, 24 h).

### RNA extraction and real-time quantitative PCR

Total RNA was isolated from cells using RNAiso Plus reagent (TaKaRa, 9109) following the manufacturer’s instructions. cDNAs were synthesized using FastKing gDNA Dispelling RT SuperMix (TIANGEN, KR118). The specific mRNAs were quantified by RT-qPCR using 2X M5 HiPer Realtime PCR mix (Mei5bio, MF015). The expression levels were calculated by the 2^−ΔΔCT^ method with β-Actin mRNA as the internal control. The primers used for RT-qPCR were as follows: β-Actin: 5’-ACTGCCGCATCCTCTTCCTC-3’ (forward) and 5’-AACCGCTCGTTGCCAATAGTG-3’ (reverse);

Hsc70: 5’-TGAGAAGTACAAGGCTGAGGATGAG-3’ (forward) and 5’-TGTTGAAGGCATAGGACTCCAGTG-3’ (reverse); Lamp2a: 5’-GCGTTTCAGATCAACACCTTTAACC-3’ (forward) and 5’-ACCGCTATGGGCACAAGGAAG-3’ (reverse).

### Western blot analysis

The cell lysates were prepared in RIPA buffer (Solarbio, R0020) supplemented with protease and phosphatase inhibitor cocktails (APExBIO, K1007, K1015), and sonicated at 25% amplitude for 3 s. After centrifugation at 15,000 g at 4ºC for 15 min, 5X SDS-PAGE sample loading buffer (Solarbio, P1040) was added to the supernatants, which were subsequently denatured at 95ºC for 5 min and immediately placed on ice to cool. Cell lysates were separated by 8% or 10% SDS-PAGE. The proteins were electrotransferred to the nitrocellulose membranes (Amersham, 10,600,002), and then blocked with 5% nonfat milk in TBS-T buffer (10 mM Tris/HCl pH 8.0, 150 mM NaCl, 0.1% Tween-20(v/v)) for 1 h at room temperature. The membrane was immunoblotted with the indicated primary antibodies for 1 h at room temperature or overnight at 4ºC. After being washed with TBS-T buffer 3 times, the membrane was incubated with secondary antibodies for 1 h at room temperature. The membranes were washed 3 times in TBS-T buffer and then visualized using a chemiluminescence (ECL) system (GE, RPN2232) or an Odyssey system (LI-COR). The relative protein amounts were determined using ImageJ software and normalized to that of β-Actin [36].

### Co-immunoprecipitation (IP) assay

GFP and GFP-HSC70 were transiently transfected into SN4741 and HEK293T cells. After transfection for 48 h, the cells were collected by cell scraping, and centrifuged at 1,000 g for 5 min. The cell pellets were lysed and Co-IP assays were performed using agarose beads conjugated anti-GFP tag mouse monoclonal antibody (Abbkine, ABT2023) according to the manufacturer’s instructions. Briefly, 1 × 10^7^ cells were lysed in 200 μL of lysis buffer (10 mM Tris/HCl pH 7.5, 150 mM NaCl, 0.5 mM EDTA, 0.5% NP40) on ice for 30 min with extensive pipetting every 10 min. The cell lysate was centrifuged at 20,000 g for 10 min at 4 °C, after which the supernatant was transferred to a precooled tube and incubated with 30 μL agarose beads at 4 °C on a rotating wheel for 2 h. The mixture was centrifuged at 2,500 g for 2 min at 4 °C, after which the supernatant was discarded. The beads were washed 3 times with dilution buffer (10 mM Tris/HCl pH 7.5, 150 mM NaCl, 0.5 mM EDTA) and then 30 μL of 2X SDS loading buffer was added. After denaturation at 95 °C for 5 min, the supernatant was subjected to standard SDS-PAGE and western blotting procedures.

### Cell proliferation assay

A total of 5,000 cells per well were plated on 96-well plates and incubated at 37℃ in a 5% CO_2_ incubator overnight. After treatment with the indicated chemicals, 10 μL of CCK-8 reagent (APExBIO, K1080) was added to each well, and the plates were incubated for another 2 h. The absorbance at 450 nm was recorded using a Synergy H1MD plate reader (BioTek).

### Immunofluorescence analysis

LDs were stained with BODIPY 493/503 according to the protocol as described previously [37]. The cells were subquently grown on sterile coverslips overnight. After treatment with the indicated chemicals, the cells were fixed in 4% formaldehyde for 15 min at room temperature and then washed with PBS three times. BODIPY 493/503 (2 μM) was added to the cells, which were subsequently incubated for 15 min. For live-cell imaging, WT and KO cells were transferred to a glass bottom cell culture dish (NEST, 801,002), after 12 h of cell attachment, the cells were washed once with PBS, and 2 ml of serum-free medium supplemented with 75 nM LysoTracker Green was added. After 40 min incubation, the medium was replaced with fresh medium without LysoTracker and confocal images were taken immediately. The cells were observed under a Zeiss LSM710 Microscope with a 63 × 1.4 DIC Plan-Apochromat oil-immersion objective.

### Statistical analysis

Densitometric analysis was performed by using ImageJ. The differences were analyzed statistically using two-tailed unpaired *t*-tests for single comparisons and one-way ANOVA for multiple comparisons in GraphPad Prism 8 software. The error bars indicate the SDs of the means of ≥ 3 independent experiments (*p < 0.05; **p < 0.01; ***p < 0.001).

## Results

### Generation of Wdr45 knockout SN4741 cell lines

**Fig. 1:**
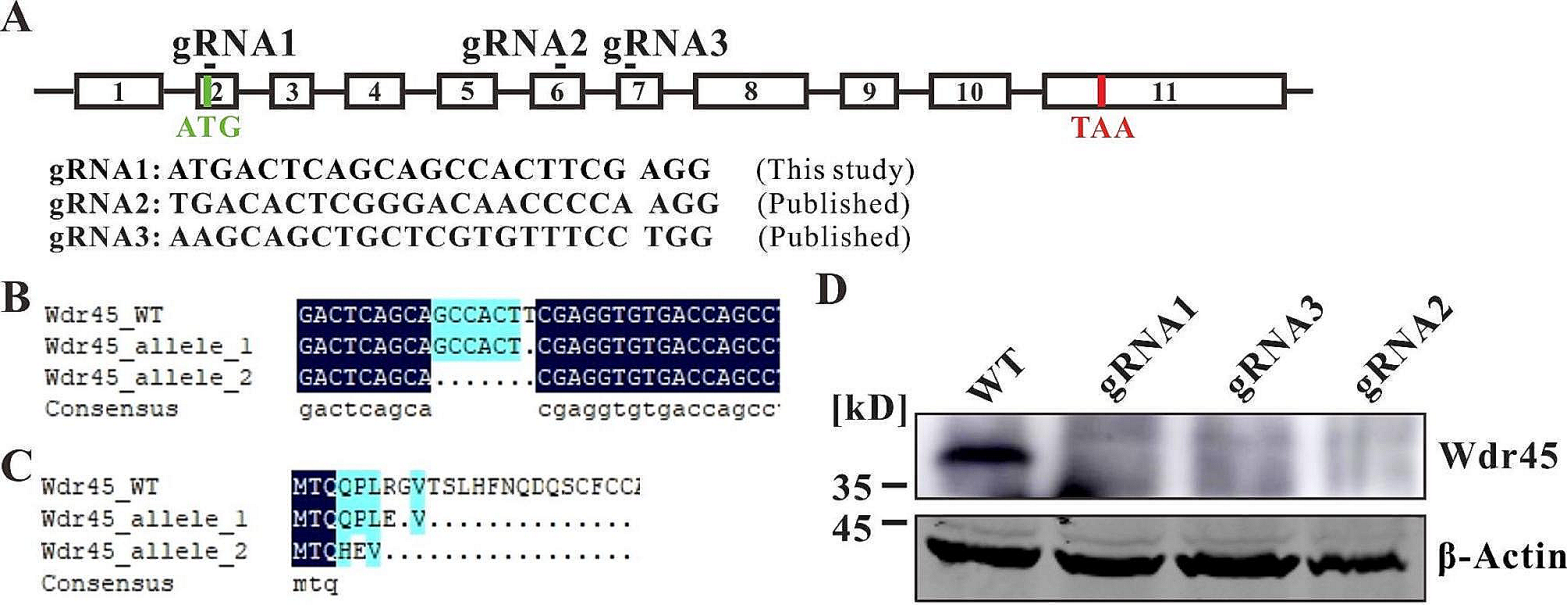
Wdr45 knockout in SN4741 cells. (**A**) The targeting site and sequence of gRNAs. (**B**) The Wdr45 knockout cell line transfected with the gRNA1 has one or seven base pair deletions in the two alleles. (**C**) Base pair deletion resulted in a frame-shift and introduced a premature stop codon. (**D**) Deletion of Wdr45 in knockout cells was confirmed using western blotting

We generated Wdr45 KO SN4741 cells using the clusters of regularly interspaced short palindromic repeats (CRISPR) associated protein 9 (CRISPR-Cas9) technique [38]. gRNA1 targets the site near the initiation codon in exon 2, gRNA2 targets the site in exon 6 and gRNA3 targets the site in exon 7 of Wdr45, respectively (Fig. 1A). The genomic DNA was extracted for polymerase chain reaction (PCR) using primers flanking the target site. DNA sequencing revealed that one, or two or seven base pairs were deleted in the two alleles of Wdr45 (Fig. 1B, S1), resulting in frame-shift mutations and the introduction of a premature stop codon (Fig. 1C, S1). Western blot analysis confirmed that Wdr45 was deleted (Fig. 1D). These results revealed that Wdr45 was inactivated in SN4741 cells and we used the Wdr45 knockout cell line that generated by the gRNA1 for further analysis.

### Wdr45 knockout impairs CMA

**Fig. 2:**
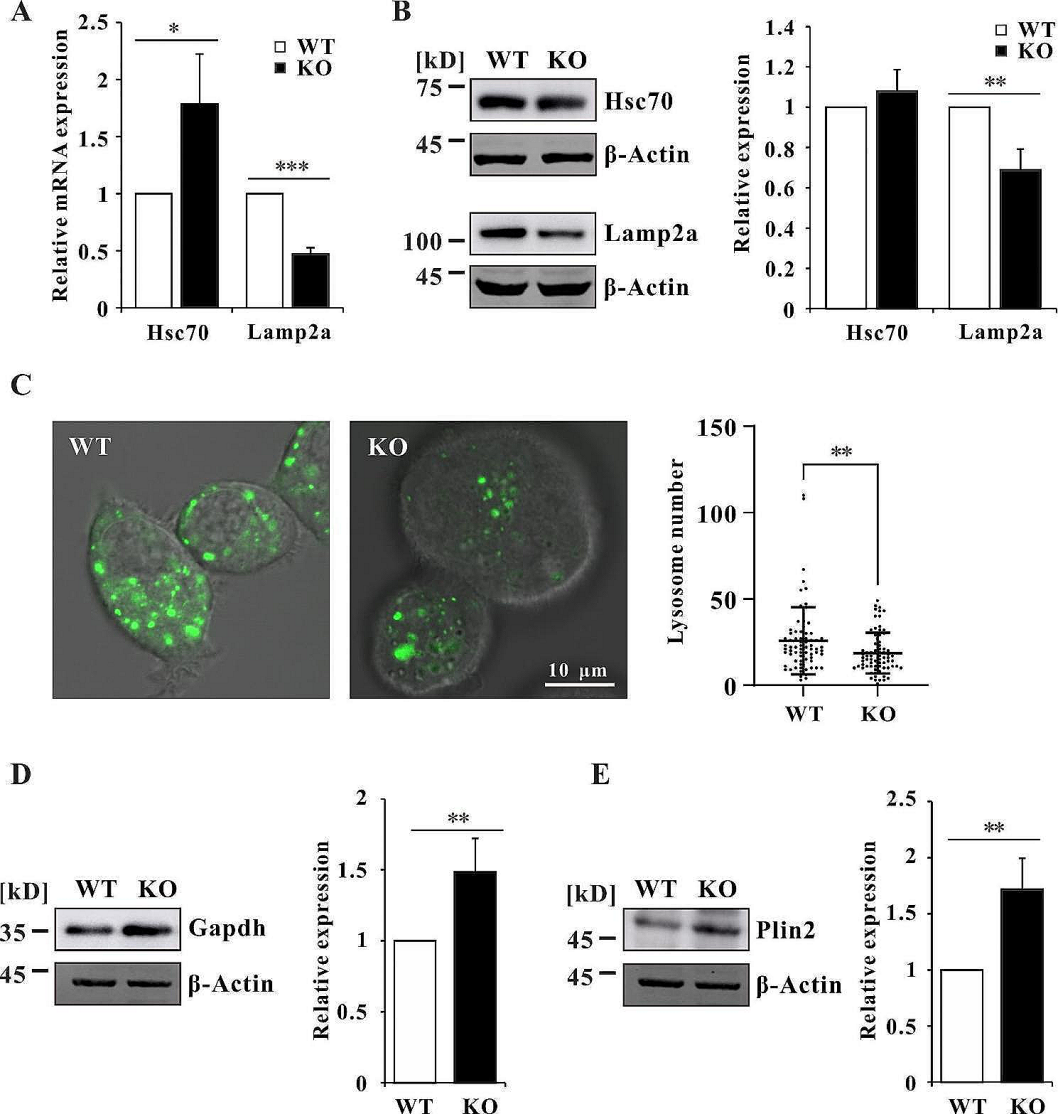
CMA activity in Wdr45 knockout cells. (**A**) The mRNA expression levels of Hsc70 and Lamp2a (*n* = 4 independent experiments). (**B**) The protein expression levels of Hsc70 and Lamp2a (*n* = 3 independent experiments). (**C**) Lysosome number in WT and KO cells. (**D**) The protein expression level of Gapdh (*n* = 4 independent experiments). (**E**) The protein expression of Plin2 (*n* = 4 independent experiments). The data are expressed as the mean ± SD; *p < 0.05, ** *p* < 0.01, *** *p* < 0.001

Both macroautophagy and CMA are involved in lipophagy. Given that Wdr45 functions in autophagosome formation, Wdr45 deficiency may impair macroautophagy. We detected the protein level of Lc3-II and p62, and found that Lc3-II was increased while p62 was decreased in Wdr45 KO cells (Figure S2A, S2B). After treatment with autophagy inhibitor Wortmannin, Lc3-II was decreased in WT cells but was unchanged in Wdr45 knockout cells (Figure S2C). Furthermore, p62 was accumulated in WT cells but not in Wdr45 knockout cells upon inhibition of autophagy (Figure S2D), suggesting that macroautophagy was impaired in Wdr45 deficiency cells. Next, we wanted to determine whether CMA is upregulated in Wdr45 knockout cells to maintain lipid homeostasis. The results showed that the mRNA expression of Hsc70 increased, while the expression of Lamp2a significantly decreased (Fig. 2A). Consistent with the qPCR results, the western blot analysis revealed that the protein level of Lamp2a was also reduced (Fig. 2B). The decreased Lamp2a indicates less lysosomes in Wdr45 knockout cells. As expected, LysoTracker staining revealed that lysosome number was significantly decreased in Wdr45 knockout cells (Fig. 2C). Furthermore, we also detected the protein levels of Gapdh and Plin2, which are substrates for CMA [29, 39]. The results showed that the expression of both of these proteins was significantly increased in Wdr45 knockout cells (Fig. 2D and E). These results clearly showed that there was no compensatory increase in CMA, and in contrast to expectations, CMA activity decreased in Wdr45 knockout cells.

### Wdr45 knockout increased Fasn thus leading to LD accumulation

**Fig. 3:**
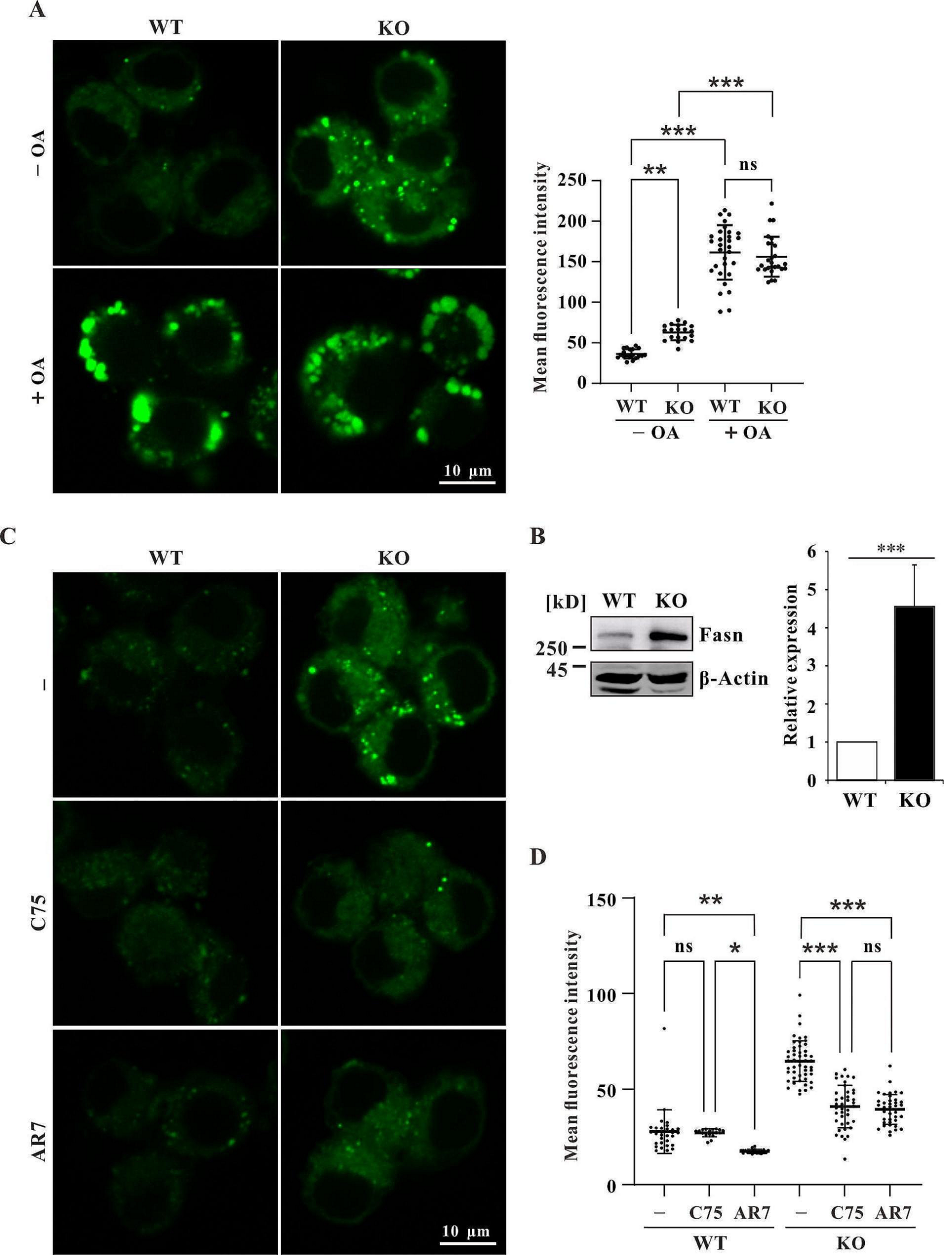
Fasn promotes LD accumulation in Wdr45 knockout cells. (**A**) LD density of WT and KO cells treated with or without 300 μM OA for 24 h. (**B**) The protein expression level of Fasn (*n* = 4 independent experiments). (**C**) LD density of WT and KO cells treated with 20 μM Fasn inhibitor C75 or 20 μM CMA activator AR7 for 24 h. The data are expressed as the mean ± SD; *p < 0.05, ** *p* < 0.01, *** *p* < 0.001

Defects in macroautophagy and CMA impair the degradation of LDs, therefore causing LD accumulation. The abundance of LDs was measured by labeling with the lipophilic fluorescent dye BODIPY 493/503. The results showed that LDs accumulated in all three Wdr45 knockout cell lines (Fig. 3A, S3). After treatment with OA to promote LD formation, the LD density was significantly greater in both the WT and KO cells (Fig. 3A).

Next, we wanted to determine whether the cells reduced the biosynthesis of lipids to protect cells from lipotoxicity. We detected the expression of fatty acid synthase (Fasn) and found that the expression of Fasn was dramatically increased (Fig. 3B). Furthermore, the LD density was significantly reduced after Fasn was inhibited with the inhibitor C75 in the KO cells but not in WT cells (Fig. 3C and D), these results suggested that Fasn promoted LD formation in KO cells but not in WT cells. However, enhancing CMA by the CMA activator AR7 in WT cells promotes the degradation of LD (Fig. 3C and D). As CMA was impaired and Plin2 was increased in KO cells, we treated KO cells with AR7 and found that the LD density decreased, as expected (Fig. 3C and D). Interestingly, the contents of LDs in C75- and AR7-treated Wdr45 knockout cells were similar, which indicated that the activation of CMA by AR7 could inhibit Fasn in mouse dopaminergic neurons in a manner similar to that of C75 (Fig. 3C). Taken together, these results suggested that increased Fasn accounts for the LD accumulation in Wdr45 knockout cells and that activation of CMA eliminates the effect of Fasn.

### Fasn can be degraded through CMA

**Fig. 4:**
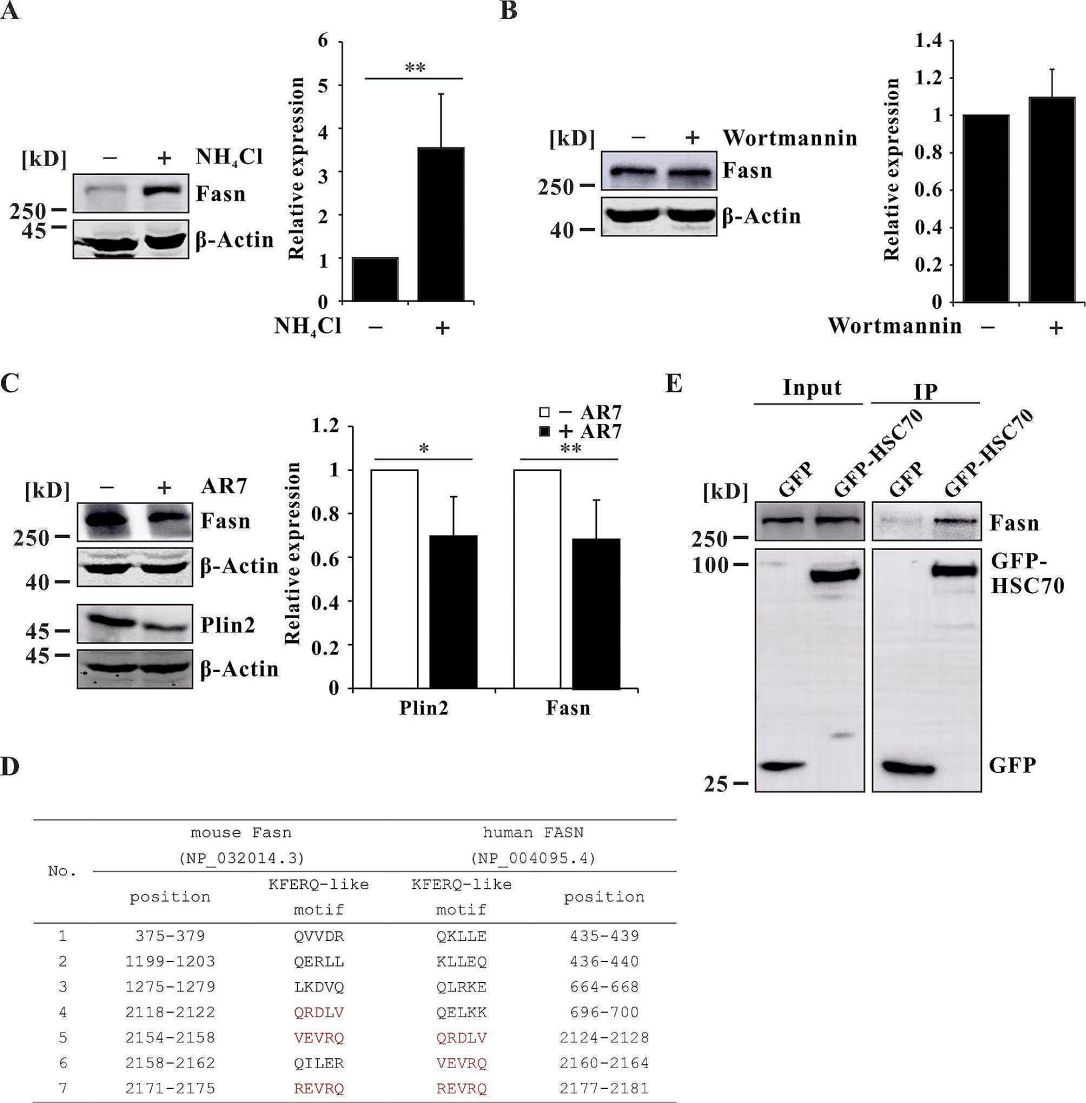
Fasn is a substrate for CMA. (**A**) The protein expression level of Fasn in WT cells treated with or without the 5 mM lysosomal inhibitor NH_4_Cl for 24 h (*n* = 4 independent experiments). (**B**) The protein expression levels of Fasn (*n* = 3 independent experiments) in WT cells treated with or without 500 nM Wortmannin for 24 h. (**C**) The protein expression levels of Fasn (*n* = 5 independent experiments) and Plin2 (*n* = 3 independent experiments) in KO cells treated with or without 20 μM AR7 for 24 h. (**D**) KFERQ-like motif prediction of mouse Fasn and human FASN. (**E**) The interaction between Fasn and HSC70 was analyzed by Co-IP. The data are expressed as the mean ± SD; *p < 0.05, ** *p* < 0.01, *** *p* < 0.001

Given that AR7 and C75 have similar effects on the downregulating LDs, we hypothesized that the activation of CMA by AR7 promotes the degradation of Fasn. To test whether Fasn can be degraded via CMA, we treated WT cells with NH_4_Cl to inhibit lysosomes and found that the protein level of Fasn was significantly elevated, suggesting that Fasn can be degraded via the lysosomal pathway (Fig. 4A). It has been proposed that FASN can be degraded via macroautophagy in human cancer cell lines [40, 41]. However, Fasn did not increase upon inhibition of macroautophagy by Wortmannin (Fig. 4B), suggesting that macroautophagy is not involved in the degradation of Fasn in mouse dopaminergic cells. We further treated the cells with the CMA activator AR7, and the protein level of Plin2 decreased, as expected (Fig. 4C). Interestingly, Fasn expression also decreased significantly upon treatment with AR7, indicating that Fasn can be degraded via CMA (Fig. 4C). Moreover, we screened the KFERQ-like motifs in Fasn using the free web-based resource KFERQ finder (https://rshine.einsteinmed.edu/) [42] and identified seven putative canonical KFERQ-like motifs, among which three were conserved in human and mouse Fasn (Fig. 4D). The interaction between Fasn/FASN and HSC70 was confirmed using Co-IP analysis (Fig. 4E, S4). These results suggested that Fasn is a substrate for CMA.

### LD accumulation reduced cell viability

**Fig. 5:**
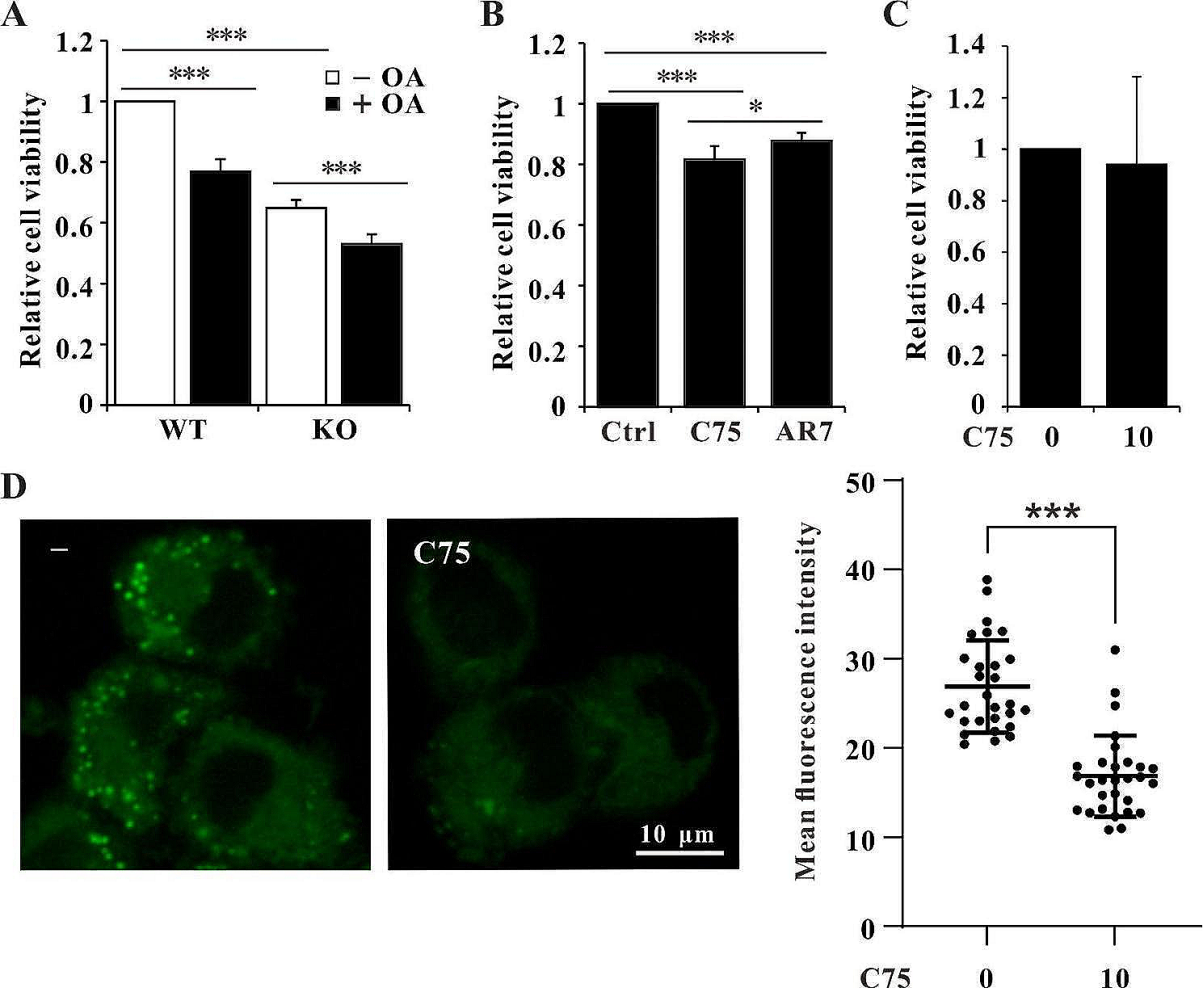
LD accumulation impaired cell proliferation. (**A**) Viability of WT and KO cells treated with or without 300 μM OA for 24 h (*n* = 4 independent experiments). (**B**). Viability of KO cells treated with 20 μM C75 or 20 μM AR7 for 24 h (*n* = 4 independent experiments). (**C**) Viability of KO cells treated with 10 μM C75 for 24 h (*n* = 3 independent experiments). (**D**) LD density of KO cells treated with 10 μM C75 for 24 h. The data are expressed as the mean ± SD; *p < 0.05, ** *p* < 0.01, *** *p* < 0.001

Whether LD accumulation is protective or toxic in mouse dopaminergic cells is unclear. We measured cell viability and found that it was reduced in Wdr45 knockout cells (Fig. 5A). Supplementation with OA to promote LD formation further decreased cell viability, indicating the toxicity of LDs to SN4741 cells (Fig. 5A). Given that LD accumulation is toxic to cells, accelerating LD breakdown or inhibiting LD formation by AR7 or C75, respectively, should rescue the viability of Wdr45-deficient cells. In contrast to our expectations, the results revealed that cell viability decreased upon treatment with AR7 or C75 in both WT and KO cells (Fig. 5B, S5). When KO cells were treated with a lower concentration of C75, cell viability did not decrease, but the LD density was reduced (Fig. 5C and D). These results suggested that Wdr45 knockout elevated Fasn, which is essential for cell survival, but excess Fasn also led to LD accumulation. Therefore, the decrease in cell viability in KO cells treated with AR7, which promotes the degradation of Fasn and LD breakdown, was milder than that in C75-treated cells (Fig. 5B).

## Discussion

BPAN was first described in 2009 [1], however, until now, the pathomechanism of BPAN has remained unknown. In 2012, Hacck et al. initially discovered that the *WDR45* mutation is the cause of BPAN [3]. As WDR45 functions in autophagy, studies have focused on the role of autophagy in BPAN. To date, numerous BPAN models, such as mouse, *Drosophila*, *Caenorhabditis elegans*, and *Dictyostelium* models, as well as human cell lines, including fibroblasts, lymphoblasts, neuroblastoma cells and induced pluripotent stem cells (iPSCs), generated from BPAN human fibroblasts, have been used to study the function of WDR45 and its homologs in the maintenance of cell homeostasis [6]. As a subtype of neurodegeneration with brain iron accumulation (NBIA), iron accumulates in the globus pallidus and substantia nigra [3]. Several studies have shown that ferritin dysregulation in WDR45 deficient cells consequently disrupts iron homeostasis [36, 43–46]. Iron accumulation may lead to ferroptosis, which promotes BPAN; however, treatment with iron chelation drugs has no benefit and can even worsen this process [47, 48]. Therefore, the roles of WDR45 and autophagy in BPAN remain to be elucidated.

Brain MRI of BPAN patients showed that iron accumulated in the globus pallidus and substantia nigra [3], and a mouse BPAN model showed a loss of dopaminergic neurons [49], indicating that dopaminergic neurons may be the most vulnerable cells to Wdr45 defects. Therefore, we deactivated Wdr45 in the mouse dopaminergic cell line SN4741 to investigate the function of Wdr45 in BPAN (Fig. 1).

Currently, PD is also considered to be a lipidopathy because lipid dyshomeostasis is one of its fundamental characteristics [50, 51]. BPAN patients share some clinical features with PD patients, therefore, we wanted to determine whether lipid homeostasis is imbalanced in BPAN patients. Lipophagy plays a pivotal role in the maintenance of lipid homeostasis [24]. Given that Wdr45 deficiency impairs macroatophagy (Figure S2), whether CMA is affected by Wdr45 deletion in mouse dopaminergic neurons is unclear. We detected the expression of two key proteins, Hsc70 and Lamp2a, and found that Lamp2a was decreased at both the mRNA and protein levels (Fig. 2A and B). Gene transcription can be regulated by protein acetylation [52]. A possibility is that acetylation of proteins such as Tfeb and histones maybe decreased therefore inhibit the expression of Lamp2a. Therefore, the decreased Lamp2a led to reduced lysosomes (Fig. 2C). Furthermore, Gapdh and Plin2, two substrates for CMA degradation, accumulated (Fig. 2D and E). These results indicated that CMA was inhibited in Wdr45 knockout cells. In our previous study, overexpression of a mutant WDR45 in HeLa cells induced ER stress, and subsequently activated CMA to accelerate the degradation of ferritin heavy chain (FTH) and glutathione peroxidase 4 (GPX4), which promote ferroptosis [36]. The differences between these two studies may be because of the different strategies used for model generation. In this study, we used CRISPR-Cas9 to introduce a premature stop codon to simulate nonsense mutations in BPAN patients, while we overexpressed the mutant WDR45 to simulate missense mutations in vivo. Several studies suggested that missense mutations in *WDR45* lead to mRNA and protein loss [44, 45, 53–55]. However, the mutant WDR45, which is expressed at a low level, can be stably expressed in HeLa cells [56], suggesting that the pathogenicity of the WDR45 missense and nonsense mutations should be different. However, the pathogenicity of WDR45 missense mutations requires further study. Nevertheless, the decrease in CMA activity caused by Wdr45 deletion remains to be addressed in future work.

The impairment of macroautophagy and CMA indicated a blockage of lipophagy; therefore, LDs accumulated in Wdr45 knockout cells (Fig. 3A, S3). When OA was added to stimulate LD formation, large puncta were detected, which indicated LDs (Fig. 3A). We further determined whether lipid synthesis also contributes to LD accumulation in mouse Wdr45 knockout dopaminergic cells. The results revealed that Fasn expression was dramatically increased (Fig. 3B) and that LD density was significantly decreased after Fasn was inhibited, indicating that lipid synthesis also contributes to LD accumulation (Fig. 3C and D). Intriguingly, the LD density was reduced to a similar level by treatment with AR7 and C75 in Wdr45 knockout cells (Fig. 3C and D), which raised the question of whether activation of CMA inhibits Fasn. It is possible that CMA activation can degrade Fasn, thereby inhibiting LD formation. Previous studies have showed that human FASN may be degraded via macroautophagy because the protein level of FASN was increased upon treatment with Bafilomycin A1 [40, 41]. Bafilomycin A1 is a well-known autophagy inhibitor that can impair lysosome function by inhibiting V-ATPase-dependent acidification and also can inhibit autophagosome-lysosome fusion [57]. We also found that mouse Fasn increased after inhibition of lysosome function (Fig. 4A). However, we did not find a significant change in mouse Fasn after autophagy inhibition by Wortmannin, an inhibitor of phosphatidylinositol-3-kinase (Fig. 4B). These results indicated that Fasn was not degraded via macroautophagy and that Fasn may be a substrate for CMA. As expected, Fasn was decreased after CMA activation (Fig. 4C). This was further confirmed by the interaction with HSC70 using Co-IP analysis (Fig. 4D and E, S4). Fasn was found to be degraded via the ubiquitin proteasome system [58]. Our results suggested that Fasn could also be degraded via CMA and promote LD accumulation in CMA-deficient mouse dopaminergic neurons.

LD accumulation is toxic to mouse dopaminergic neurons, as evidenced by the decreased cell viability upon treatment with OA (Fig. 5A). Surprisingly, Fasn inhibition with C75 did not rescue cell viability but even decreased cell viability (Fig. 5B). These results suggested that Fasn should have other functions that are essential for cell survival. Fasn is the terminal enzyme in fatty acid (FA) synthesis, and FAs are components of cell membrane phospholipids [59]. In many human tumors, Fasn is increased to provide enough FAs to support the rapid proliferation of cancer cells [31]. Complete suppression of Fasn impaired FA biosynthesis, which led to a shortage of FAs to support cell survival. Therefore, inhibition or degradation of Fasn reduce the cell viability in WT cells (Figure S5). We partially inhibited Fasn using a low concentration of C75 in Wdr45 knockout cells, and the results showed that cell viability was not impaired but that LD accumulation was reduced (Fig. 5C). These results suggested that Wdr45 deletion impaired lipophagy, therefore, blocking LD turnover; thus, the cells upregulated Fasn to provide enough FAs for consumption. However, excess FAs promote LD formation, which is toxic to the cell. AR7 treatment promoted Fasn degradation, which led to decreased FA formation; however, it also promoted lipophagy, which is beneficial to the cell. Therefore, the viability of the AR7-treated cells decreased, but the decrease was milder than that in the C75-treated cells (Fig. 5B).

## Conclusions

**Fig. 6:**
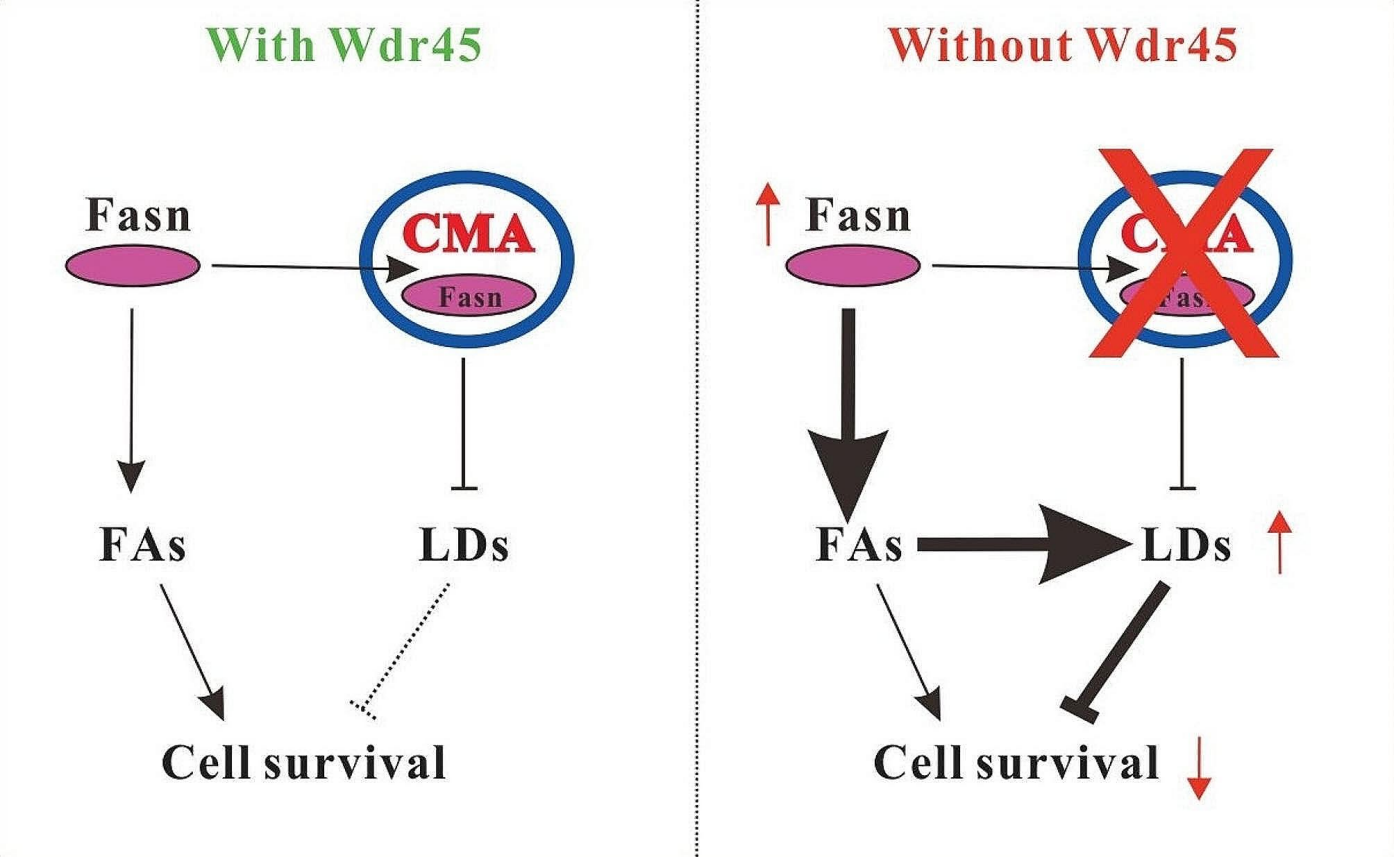
Schematic of the mechanism by which excess Fasn promotes LD accumulation in Wdr45 deficient cells. In normal cells, CMA degrades Fasn and LDs, thus suppressing LD accumulation, which is toxic to mouse dopaminergic neurons SN471. However, deletion of Wdr45 impaired CMA. Neither Fasn nor LDs can be degraded; thus, excess Fasn promotes LD formation. Increased LD formation and impaired LD breakdown inhibit cell proliferation which may contribute to the progression of BPAN

Our study revealed that under normal physiological conditions, CMA regulates the homeostasis of Fasn and LDs, which promotes cell survival. Wdr45 deletion led to increased Fasn expression via impairment of CMA, which resulted in increased LD formation but decreased LD breakdown. Consequently, LDs accumulate, and cell viability decreases, which may contribute to the progression of BPAN (Fig. 6).

### Electronic supplementary material

Below is the link to the electronic supplementary material.

Supplementary Material 1

## Data Availability

No datasets were generated or analysed during the current study.
