# Supplementary material for: Lipid droplet accumulation in Wdr45-deficient cells caused by impairment of chaperone-mediated autophagic degradation of Fasn
Source: Lipids Health Dis. 2024 Mar 28;23:91. doi: 10.1186/s12944-024-02088-y (PMC10976834; doi:10.1186/s12944-024-02088-y)

Figure S1: The sequencing chromatograms of Wdr45 knockout cells. (A) The sequencing results of Wdr45 knockout cells generated by gRNA1. (B) The sequencing results of Wdr45 knockout cells generated by gRNA2. (C) The sequencing results of Wdr45 knockout cells generated by gRNA3.


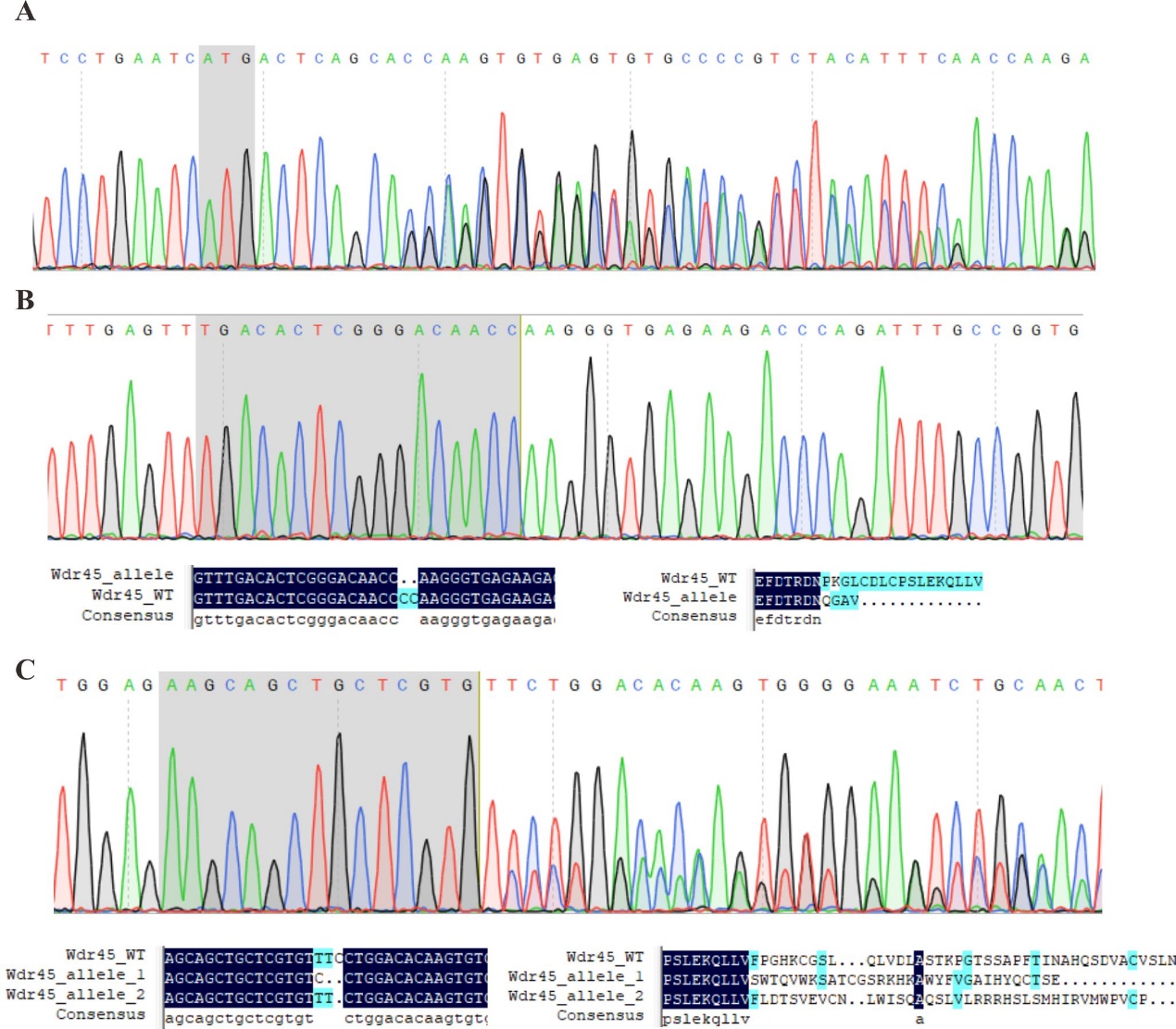


Figure S2: Macroautophagy was impaired in KO cells. (A) The protein expression of Lc3 in WT and KO cells (n=3 independent experiments). (B) The protein expression of p62 in WT and KO cells (n=4 independent experiments). (C) The protein expression of Lc3 in WT and KO cells after treatment with Wortmannin (500 nM, 24 h) (n=3 independent experiments). (D) The protein expression of p62 in WT and KO cells after treatment with Wortmannin (500 nM, 24 h) (n=3 independent experiments). The data are expressed as the mean ± SD; **p*< 0.05, ** *p* < 0.01, *** *p* < 0.001.


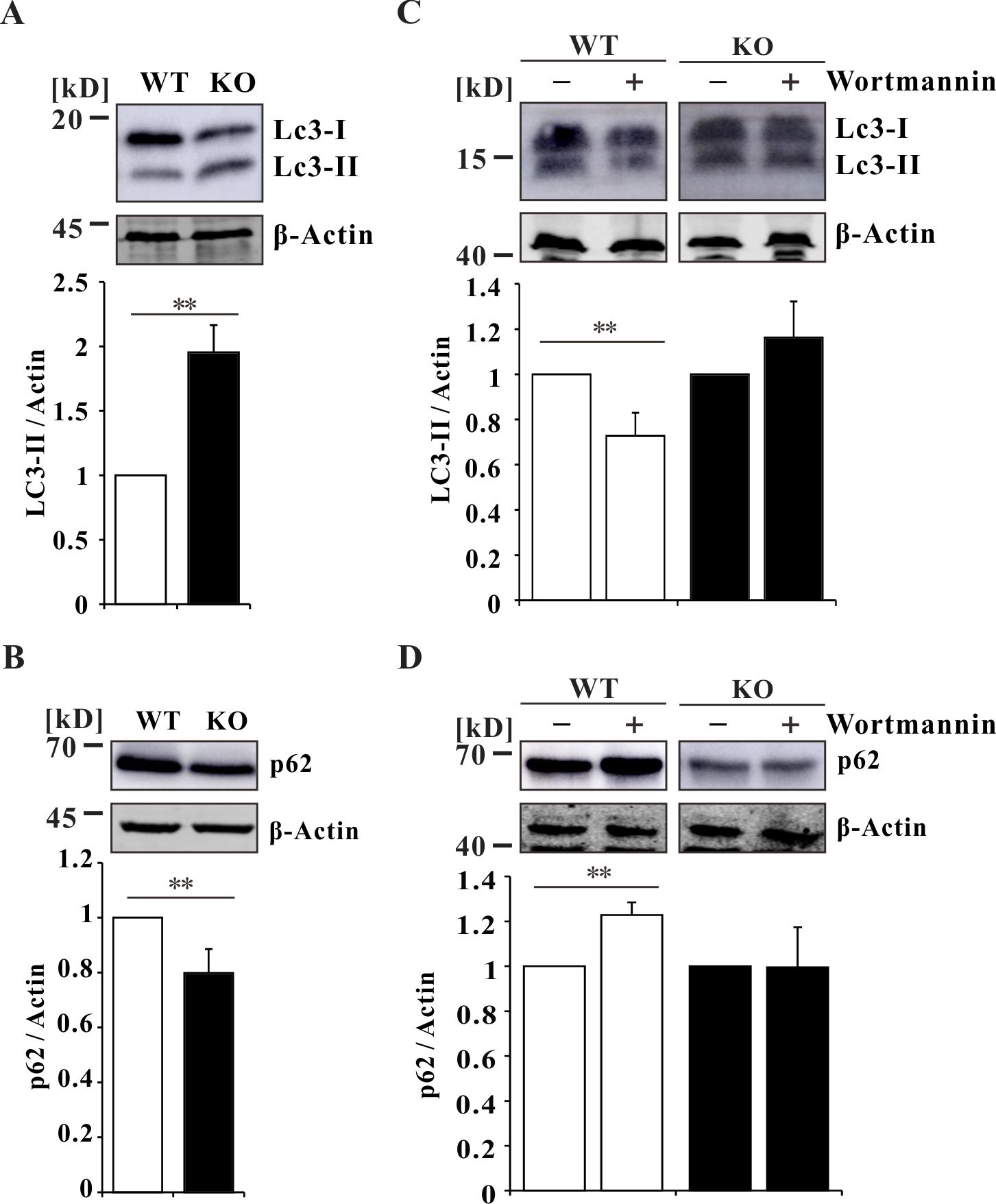


Figure S3. LD density in other 2 KO cell lines. The data are expressed as the mean ± SD; **p*< 0.05, ** *p* < 0.01, *** *p* < 0.001.


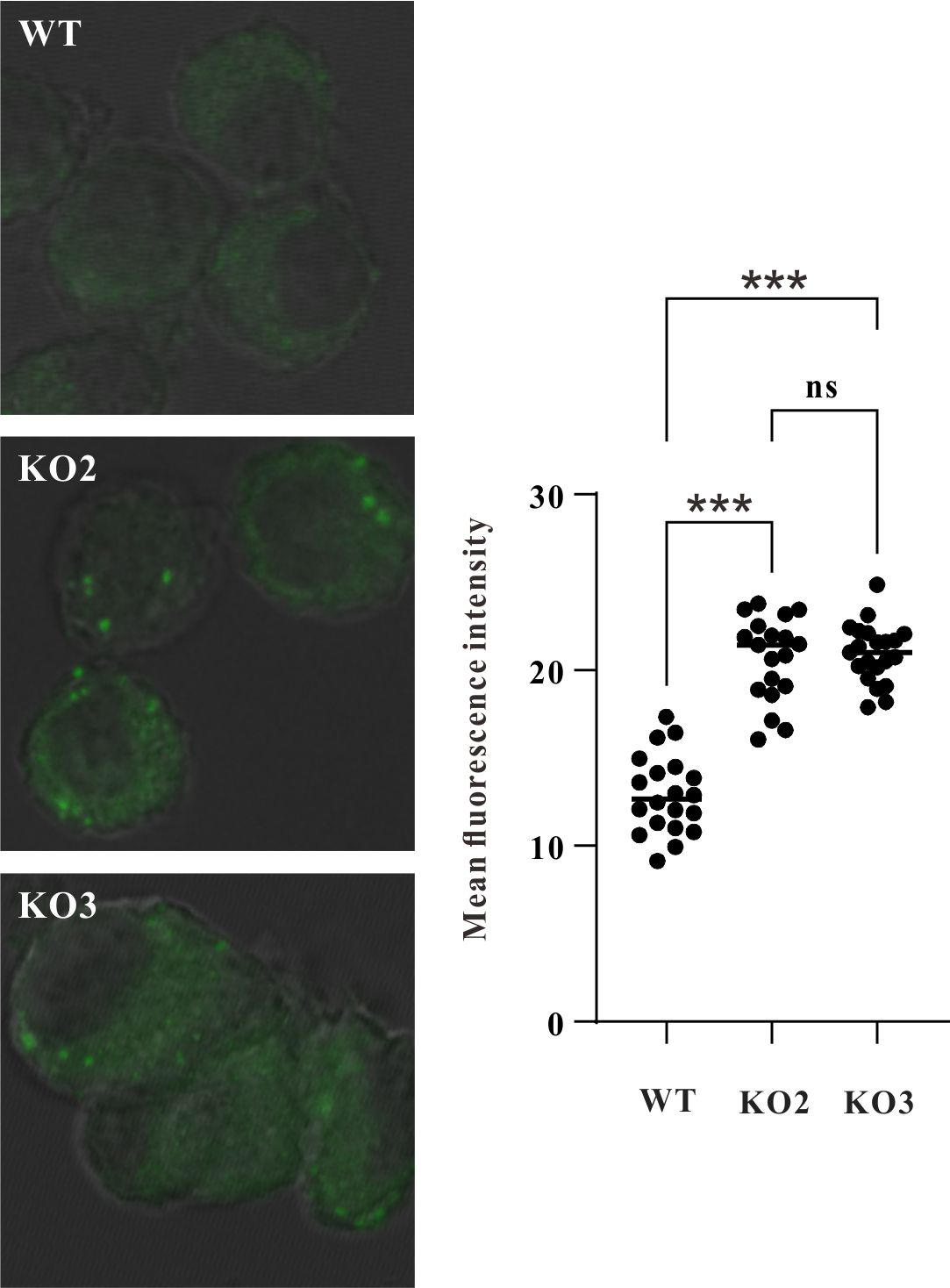


Figure S4. Human FASN interacted with HSC70. The interaction between human FASN and HSC70 were analyzed by Co-IP.


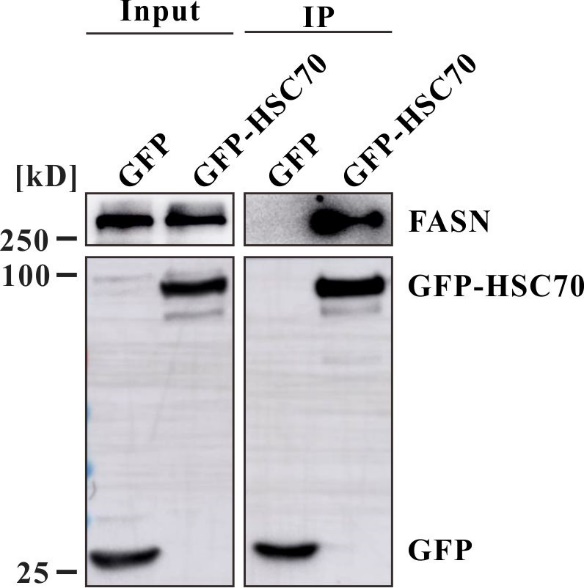


Figure S5. Viability of WT cells treated with 20 μM C75 or 20 μM AR7 for 24 h (n=3 independent experiments). The data are expressed as the mean ± SD; **p*< 0.05, ** *p* < 0.01, *** *p* < 0.001.


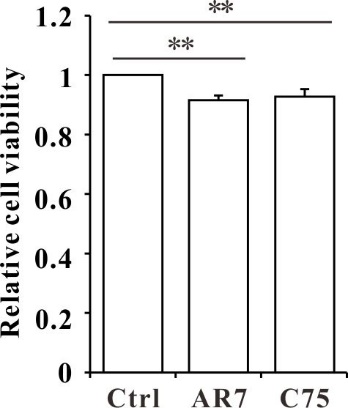

Supplement: Supplementary file 1 — Supplementary Material 1 [file 12944_2024_2088_MOESM1_ESM.docx]
